# Values interact with psychological distance and eco-anxiety to promote climate engagement: insights from two experimental studies

**DOI:** 10.3389/fpsyg.2025.1646889

**Published:** 2025-09-19

**Authors:** Hanwen Zhang, Wen Xu, Michelle McCauley

**Affiliations:** ^1^Stanford Center on China’s Economy and Institutions, Freeman Spogli Institute for International Studies, Stanford University, Stanford, CA, United States; ^2^Department of Psychology, Middlebury College, Middlebury, VT, United States; ^3^Khoury College of Computer Sciences, Northeastern University, Boston, MA, United States

**Keywords:** value orientation, psychological distance, eco-anxiety, eco-emotions, climate change

## Abstract

The effectiveness of encouraging engagement with climate change through reducing its psychological distance has been increasingly called into question. Through two experimental studies, we examine how value orientation interacts with proximal information and eco-anxiety to affect climate engagement. Study 1 (N = 472) tested how exposure to spatially proximal versus distal messaging conditions affected psychological distance to climate change and subsequent climate risk perception, policy support, and mitigation intention. We found that spatial messaging conditions did not significantly affect psychological distance or climate engagement. However, both self-transcendence values and proximal distance predicted more climate engagement. Additionally, the positive association between proximal psychological distance and pro-environmental behavioral intention was stronger among individuals with higher self-transcendence and weaker among individuals with higher self-enhancement. Study 2 (N = 414) examined whether a self-reflective writing task to invoke eco-anxiety is more effective than proximal messaging at increasing climate engagement. We found that the writing task was more effective than proximal messaging at eliciting eco-anxiety, which positively predicted risk perception, policy support, information sharing intention, and effortful mitigation behavior. Path analysis reveals that stronger self-transcendence values not only directly predicted more climate engagement but also predicted higher eco-anxiety in response to interventions. Collectively, our results suggest that elicitation of eco-anxiety could be a superior strategy for increasing engagement with climate change than exposure to information about its local impacts.

## 1 Introduction

Climate change is intensifying at unprecedented rates. Researchers have emphasized the need to limit the global temperature increase at, or below, 1.5°C to prevent severe ecosystem damage which will endanger human life (Slade et al., 2022). Research has demonstrated the urgency of individual action to mitigate climate change (Wynes and Nicholas, 2017). However, despite growing recognition of the negative impacts of climate change, individual actions to mitigate climate change have been limited. While an estimated 64% of U. S. adults report that climate change affects their local communities, support for renewable energy has dipped since 2020 and only about 25% see limiting their personal carbon footprint as a high priority (Tyson and Kennedy, 2024). Given the urgency, understanding the cognitive and emotional processes that shape perception of climate change and engagement in mitigation behaviors is critically important. Through two experimental studies, we examine how individual value orientation interacts with the psychological distance of climate change and anxiety about it to affect pro-environmental intentions and behavior. In the following sections, we review prior research on how psychological distance, value orientation, and anxiety about climate change influence climate engagement.

## 2 Literature review

### 2.1 Psychological distance and climate engagement

People are more likely to believe that others will be harmed by climate change than themselves (Keller et al., 2022). In the U. S., this bias has persisted despite that communities across the country have experienced considerable impacts of climate change (Jay et al., 2023). Research has proposed that reducing the psychological distance of climate change by rendering it as local and personal is key to increasing climate engagement. Construal Level Theory (henceforth, CLT) scholars define psychological distance as the subjective experience that something is close or far away from the self, here, and now (Trope and Liberman, 2010). Psychological distance comprises four dimensions: Spatial distance and temporal distance are the extent to which an event feels proximal in space and in time, respectively. Hypothetical distance is the perceived probability of an event and social distance refers to the extent to which one believes an event affects groups of people similar to oneself. Events that are psychologically closer are thought of more concretely and are thus more likely to elicit engagement. Many scholars have suggested that these dimensions are predictive of concern and engagement with environmental issues (Tang and Chooi, 2023).

Contrary to this assumption, however, there is mixed empirical evidence for the effect of psychological distance on climate engagement. On the positive, studies have supported the associations between closer spatial, temporal, hypothetical, and social distances to climate change and stronger personal intentions of mitigation behavior (Sacchi et al., 2016; Spence et al., 2012). There is also experimental evidence that proximally framed messages increased climate mitigation intentions and support for pro-mitigation policies (Jones et al., 2017; Loy and Spence, 2020). Unfortunately, a growing body of studies shows that reducing one’s psychological distance to climate change does not always increase engagement (for a full review, see Maiella et al., 2020). For example, Brügger et al. (2015) examined how perceiving climate change as either a spatially proximal (local) or distant risk predicted the intentions for personal mitigation behavior and supporting pro-climate policies. Contrary to CLT, they found that a distant risk perception better predicted support for mitigation and adaptation policies than a proximal one, and that the strength of the associations between personal mitigation behavior intentions and proximal and distant risk perceptions were similar. Studies that used messaging conditions to manipulate spatial distance (Schuldt et al., 2018) and temporal distance (Rickard et al., 2016) also found that reduced spatial and temporal distance to climate change did not increase engagement.

Given the sustained increase in severe weather events and climate disasters that impacted the U. S. in the past decade (Smith, 2024), there could be a substantial floor effect in spatial distance because many people have recently experienced climate disasters close to home. To better understand how spatial psychological distance interacts with value orientation to affect climate engagement, the effect of spatial-distance-based messaging needs to be assessed through verifiable measures.

In addition, since psychologically proximal events are processed more analytically and effortfully, they are also more likely to be met with defensive or motivated skepticism. Messages that describe environmental issues as proximal can lead to backlash and reduce engagement if the information provided is incongruent with one’s worldview. Roh et al. (2015) found that framing the Lyme disease epidemic as temporally proximal and anthropogenic, a framing in conflict with the conservative view which attributes environmental issues to environmental factors (McCright and Dunlap, 2011), decreased Republicans’ acknowledgment of anthropogenic attribution of the disease and their intention of conservation behaviors. Later studies that manipulated psychological distance through messaging conditions found that psychological proximity to climate change can predict a stronger backlash to worldview-incongruent messages. Using visual tasks that induced different levels of spatial psychological distance, Yang et al. (2021) demonstrated that a proximal cue increased climate risk perception among liberals and moderates but not conservatives. Similarly, Rickard et al. (2016) found that U. S. conservatives reported the highest level of climate policy support when reading a passage that described climate change as spatially proximal but temporally distal, suggesting that temporally distant framings lessened the backlash to the worldview-incongruent message. Besides political leanings, growing evidence suggests that value orientation also moderates the effect of psychological distance: For example, through presenting residents of Vermont, U. S. with information of a flood that took place either in Vermont (proximal condition) or in Pakistan (distal condition), Schoenefeld and McCauley (2016) found that individuals with stronger self-enhancement values were less willing to engage in pro-environmental behavior when receiving local information than when receiving distal or no information, while those with stronger self-transcendent values reported stronger intentions for pro-environmental behaviors regardless of distance framing.

### 2.2 Value orientation and climate engagement

The diverging reactions of individuals with different value orientations to proximally framed messages suggest that value orientation is crucial for understanding the varying effects of psychological distance on climate engagement (Schoenefeld and McCauley, 2016). Values, defined as beliefs that transcend specific situations to determine desirable end states and guide behavior (Schwartz, 1994), influence how accessible each of these goals are in the decision-making process. Previous works have generally identified two value dimensions that influence environmental attitudes and behavior (Schwartz et al., 2012). Self-transcendence values emphasize concern for the welfare and interests of others, including social-altruistic values, which regard benefits of other people and the society as important, and biospheric values, which encourage individuals to act in ways that favor the natural environment and non-human species (Stern and Dietz, 1994). On the other hand, self-enhancement values emphasize pursuit of one’s own interests, hedonism, and relative success and dominance over others. They include egoistic and hedonic values, which guide individuals to benefit their own interests and prioritize personal gain and enjoyment like cost and comfort. A substantial body of research has shown that self-enhancement values inhibit pro-environmental behaviors while self-transcendence values promote them (Nordlund and Garvill, 2003; Schultz et al., 2005; Steg et al., 2014). Specifically, self-enhancement values were found to be positively correlated with climate skepticism (Grapsas et al., 2023) and negatively correlated with personal responsibility and self-reported energy saving behavior (Boto-García and Bucciol, 2020). In addition, individuals with stronger self-enhancement values showed weaker intentions for pro-environmental actions when exposed to the local impacts of climate change than to its distant impacts or no information at all (Schoenefeld and McCauley, 2016), suggesting that self-enhancement values may amplify the backlash to worldview-incongruent messages.

However, the association between self-enhancement and inaction towards climate change has been increasingly contested. For example, Bergquist et al. (2022) found that the negative correlation between self-enhancement and support for mitigation policies is weak, proposing that egocentric values may not be a strong barrier for accepting policies aimed at mitigating climate change. In support of this conjecture, research that examined pro-environmental political organizing found that egoistic values, but not altruistic or biospheric values, predicted pro-environmental lobbying (Sloot et al., 2018). Within egoistic values, valuing ambition and influence predicted lobbying whereas valuing power and material wealth did not, suggesting that the egoistic goal of achieving one’s objectives and influencing others to support one’s cause can encourage pro-environmental behavior in the political arena.

These findings point to the previously overlooked potential of associating pro-environmental behavior with personal success and influence to encourage climate engagement among populations with strong self-enhancement. To our knowledge, few works have examined the motivating effect of self-enhancement on pro-environmental behaviors besides lobbying, especially everyday emission-reducing behavior. This gap in research is mirrored in practice, as many climate messaging campaigns associate mitigation exclusively with self-transcendence and altruism while few highlight egoistic motivations to engage in conservation (Corner et al., 2014). As a result, those who strongly endorse self-enhancement values could view climate action as an implicit attack on their values. Given this lack of attention to climate communication strategies that call on self-enhancement values in both academic and applied contexts, the present study aims to explore the effectiveness of motivating pro-environmental behavior through priming individuals to the negative consequences of climate change on their personal gain and well-being.

### 2.3 Eco-anxiety and climate engagement

Besides the interaction of psychological distance and value orientation, a distinct but related body of research has shown that eco-anxiety, defined as future-oriented apprehension towards the impacts of climate change (Clayton and Karazsia, 2020), affects climate engagement. Anxiety in response to climate change has been widespread and well documented in psychology literature (Ogunbode et al., 2022; Rozuel and Bellehumeur, 2022). A survey on public opinion in 31 countries revealed that the majority in each country reported being “very” or “somewhat” worried about climate change (Leiserowitz et al., 2021). However, researchers have disagreed on the behavioral consequences of eco-anxiety.

From a functional perspective, anxiety helps individuals anticipate and prepare for potentially harmful situations via sustained alertness and hypervigilance to threats (Parsafar and Davis, 2018). Neurological studies also suggest that anxiety corresponds to a system of defense related to approaching sources of threat (McNaughton and Corr, 2004), which should motivate active coping behavior. In an experiment where the fear of global warming and the strength of persuasive messages for pro-environmental consumption were manipulated (Meijnders et al., 2001), strong messages led to significantly more favorable attitudes and intentions for pro-environmental consumption than weak messages only when people experienced moderate fear, and greater fear predicted more message-relevant cognitive responses. Both findings suggest that fear of climate change led to greater systematic processing and uptake of messages. A later two-wave longitudinal survey study (Pavani et al., 2023) also found that individuals’ experience of eco-anxiety at baseline was positively correlated with self-reported engagement in pro-environmental behaviors 3 weeks later, even when controlling for ecological identities and personality.

On the other hand, the high physiological reactivity as a result of anxiety often leads to a generalized worry, which can cause avoidance behavior to mitigate this uncomfortable response (Paulus and Stein, 2006). Eco-anxiety can cause avoidance of information which increases feelings of threat (Shepherd and Kay, 2012) and lead to helplessness which inhibits people from acting in response to climate change (Albrecht, 2011). While fear-driven representations of climate change capture people’s attention and facilitate information processing, they do not motivate personal engagement with the issue and may instead trigger avoidance behavior, such as denial (O’Neill and Nicholson-Cole, 2009). Recent correlational research has found that higher levels of eco-anxiety were unrelated to personal emission-reducing behaviors (Goldwert et al., 2023) and were even associated with lower engagement in collective environmental campaigns (Stanley et al., 2021), suggesting an inhibitory effect of eco-anxiety on climate engagement.

To explain these divergent findings, potential interactions between eco-anxiety and value orientation on climate engagement warrant investigation. Bouman et al. (2020) have documented that eco-anxiety is only positively associated with mitigation behavior when individuals have strong feelings of personal responsibility for climate change. It is possible that strong self-transcendence values invoke a sense of personal responsibility so that high eco-anxiety leads to climate engagement, whereas self-enhancement values encourage avoidance when one is experiencing high eco-anxiety. However, because previous studies on eco-anxiety typically conceptualized eco-anxiety as a relatively immutable trait (e.g., Bouman et al., 2020; Goldwert et al., 2023; Pavani et al., 2023; Stanley et al., 2021), causal inferences between eco-anxiety and climate engagement have been difficult to establish. Considering this shortcoming, we draw on studies that used elicitation devices to invoke incidental affective responses (Lu and Schuldt, 2015) to examine whether messaging conditions can influence climate engagement through eliciting eco-anxiety, and whether value orientation mediates their effect.

**Figure 1:**
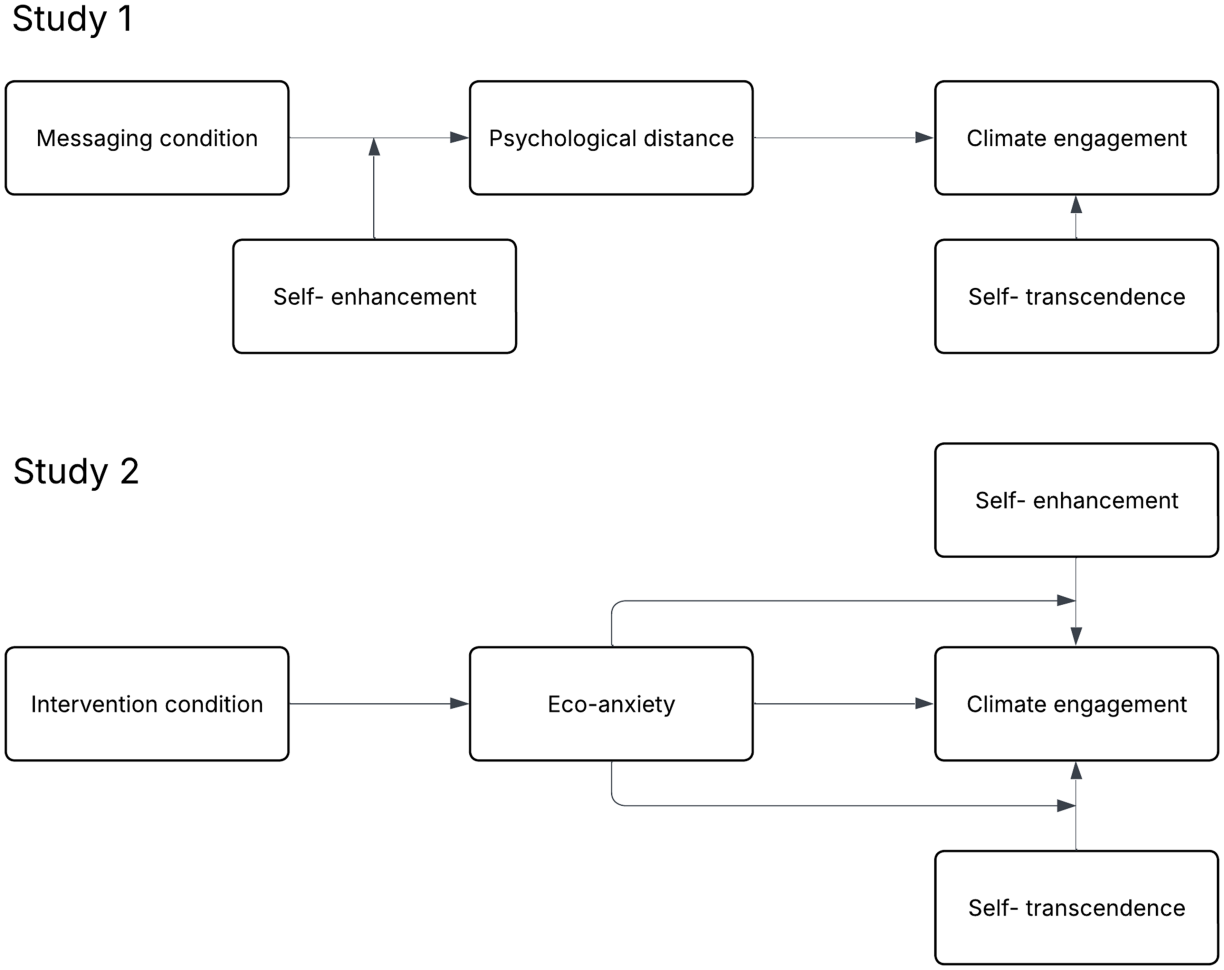
Conceptual diagram of Study 1 and Study 2.

We conducted two experimental studies to examine how value orientation interacts with proximal information and eco-anxiety to affect climate engagement, respectively (see Figure 1 for conceptual diagrams of the studies). Study 1 tested how messages that described the impact of climate change as spatially proximal versus distal affected recipients’ psychological distance to climate change and subsequent climate risk perception, policy support, and mitigation intentions. We further examined whether their value orientations moderated the link between psychological distance and climate engagement. We tested three hypotheses. First, we hypothesized that messaging conditions affect psychological distance to climate change, such that participants exposed to proximal messaging would report climate change as spatially closer than participants in the control condition, who would in turn report climate change as spatially closer than those exposed to distal messaging. Second, we hypothesized that stronger self-transcendence values predict more climate engagement regardless of messaging conditions. Third, considering the documented association between self-enhancement values and backlash to worldview-incongruent messages, we hypothesize that stronger self-enhancement values predict less climate engagement when participants are exposed to proximal information, while there will be no significant association between self-enhancement and climate engagement when participants are exposed to distal messaging or the control condition.

Study 2 tested whether prompting individuals to recall an experience of climate change that made them feel anxious affected their level of eco-anxiety and subsequent climate risk perception and policy support. We additionally measured actual mitigation behavior instead of intentions in Study 2 using a version of the Work for the Environmental Protection Task (Lange and Dewitte, 2022). We also examined whether individuals’ value orientation mediated the link between eco-anxiety and climate engagement. We tested three hypotheses. First, we hypothesized that intervention affects eco-anxiety, such that participants given a self-reflective task designed to elicit eco-anxiety would report higher eco-anxiety than participants exposed to proximal messaging, who would in turn report higher eco-anxiety than participants in the control condition. Second, we hypothesized that strong self-transcendence values predict more climate engagement, and that this association is stronger when participants have higher eco-anxiety. Third, we hypothesized that stronger self-enhancement values predict lower climate engagement among individuals with higher eco-anxiety but not among individuals with lower eco-anxiety.

## 3 Study 1: messaging and psychological distance

We preregistered the methods and analysis plan for Study 1 on the Open Science Framework (OSF).^1^

### 3.1 Materials and methods

Both studies were carried out in accordance with the recommendations of the Institutional Review Board of Middlebury College, with written informed consent from all participants. All data collected from participants have been de-identified. The research is not federally funded, regulated by the FDA, or conducted under a Certificate of Confidentiality. The protocol for Study 1 was approved by the Institutional Review Board of Middlebury College (IRB #147). Participants were treated in accordance with APA ethical guidelines.

#### 3.1.1 Participants

We used CloudResearch to recruit 847 adults who were living in Texas, USA in March 2023. We targeted the Texas population because this population is more politically diverse and thus would address the limitation of an overly liberal sample in Schoenefeld and McCauley (2016). Additionally, participants from Texas are more likely to have experienced impacts of climate change recently, which we anticipated would strengthen the effects of the proximal messages describing a recent climate disaster in Texas. Participants received small amounts of cash, reward points, or gift cards depending on the contractual agreement between CloudResearch and the platform they used to solicit participation. Out of the 847 participants recruited, only 777 provided complete survey data. We further excluded 305 participants who failed either one of two attention checks. The first attention check was an open-ended question after the climate messages to evaluate whether participants have carefully read the passage: “If you were to describe these paragraphs in your own words to a friend or family member, what would you say?” We excluded participants whose answers were irrelevant to the passages or too vague to indicate that they read the passage carefully (e.g., single-word answers such as “weather” and “terrible”). The second check was a multiple-choice item embedded in the Psychological Distance scale to evaluate attention while answering the scales: “Nine plus seven is equal to 16.” Participants who did not select “Strongly agree” were considered as failing the check. On average, participants completed the survey in 12.6 min.

#### 3.1.2 Measures

##### 3.1.2.1 Value orientation

Participants’ value orientation was measured using a 15-item version of the Schwartz Value Survey (SVS) from Stern et al. (1998) (see Supplementary material 2.1). The scale measures the four value orientations theorized by Schwartz et al. (2012) and further divides the self-transcendence orientation into altruistic and biospheric value subscales. Each subscale consists of three items. Participants rated each item on a seven-point scale to indicate how important it was as a guiding principle for them, where −1 = *opposed to my values*, 0 = *not important*, and 5 = *extremely important*. Example items include “a world at peace, free of war and conflict” and “authority, the right to lead or command.” The self-transcendence subscale showed high internal consistency (α = 0.86), while the self-enhancement subscale demonstrated acceptable internal consistency (α = 0.69).

##### 3.1.2.2 Psychological distance

Participants’ perceived distance of climate change was measured with eight items derived from Wang et al. (2019) (α = 0.85; see Supplementary material 2.2). Four of the items measured the spatial distance of climate change and the other four measured social distance. Participants indicated the extent to which they agreed with each item on a five-point scale where 1 = *strongly disagree* and 5 = *strongly agree*. One example of a spatial distance item is, “serious effects of climate change will mostly occur in areas far away from here.” We additionally embedded one attention check item in the scale, “Nine plus seven is equal to 16.” Participants who responded with any option other than “strongly agree” were considered as having failed the attention check.

##### 3.1.2.3 Risk perception

Risk perception of climate change was measured with four items on a seven-point scale (see Supplementary material 2.3). The first three items were adapted from the risk perception scale used by Chu and Yang (2020): Participants indicated their level of concern about climate change, their believed severity of its impact, and their certainty that such impact would occur (α = 0.93). We added a fourth item which asked participants to indicate how important they think climate change is when compared to other political and social issues. We computed a single indicator of risk perception by averaging scores across all items. A higher average score indicates higher perceived risk of climate change. The adapted scale had high internal consistency (α = 0.94).

##### 3.1.2.4 Mitigation intention

Participants’ intention to mitigate climate change was measured using an adapted version of the pro-environmental behavior scale from Wang et al. (2019) (α = 0.69; see Supplementary material 2.4), which asked participants to indicate how likely they were to practice the pro-environmental behavior described by each item, on a five-point scale where 1 = *very unlikely* and 5 = *very likely.* Each item described a choice between a pro-environmental action and an alternative that requires less time, money, effort, or compromises in social relationships. An example item is, “Catch a bus somewhere for 20 min, rather than driving there for 5 min.” This forced choice design could reduce the ceiling effect that many pro-environmental behavior scales have. We selected six items that described trade-offs in time, money, and effort from the original scale and adapted them according to the living conditions and environmental literacy of our sample. We added one item that captured water saving behavior: “Take a 5-min shower instead of a 10-min shower to cut down on water use.” We computed a single indicator of mitigation intention by averaging scores across all items. A higher average score indicates higher intention to take personal mitigation actions. The adapted scale had relatively low internal consistency (α = 0.44).

##### 3.1.2.5 Policy support

Climate policy support was measured using four items derived from Schoenefeld and McCauley (2016) (see Supplementary material 2.5). Participants were asked to indicate the extent to which they support four different types of pro-environmental policies. We adapted and simplified the original wording of the items to improve readability for participants with varying levels of literacy. We similarly computed a single indicator of policy support by averaging scores across all items. A higher average score indicates higher support for pro-environmental policies. The adapted scale had high internal consistency (α = 0.85).

##### 3.1.2.6 Efficacy perception

We used the Efficacy Perception scale (α = 0.93) developed by Chu and Yang (2020) based on previous works on self-efficacy and climate activism (Kellstedt et al., 2008; Morton et al., 2011) to measure participants’ perceived efficacy of climate mitigation actions (see Supplementary material 2.6). On a five-point scale where 1 = *strongly disagree* and 5 = *strongly agree*, participants indicated the extent to which they agreed with four items. Two of the items, including “I believe my actions can have a beneficial influence on climate change,” measured self-efficacy. The other two items, including “Climate change can be averted by mobilizing collective effort,” measured collective efficacy. We computed a single indicator of efficacy perception by averaging scores across all items. Higher scores indicate higher efficacy perception.

##### 3.1.2.7 Personal experience of hurricanes

Research supports the associations between climate change and many weather-related disasters (Visser et al., 2014); This includes the link between increasing late summer and early fall sea surface temperature and powerful Atlantic hurricanes (Elsner, 2006; Holland and Bruyère, 2014) which frequently affect Texas. We asked whether the participants have personally experienced a hurricane with one item: “I have personally experienced a hurricane.” Participants who indicated that they have experienced a hurricane were given two follow-up items measuring the perceived damage of the hurricane on their personal property and community, on a five-point scale where 1 = *strongly disagree* and 5 = *strongly agree.*

##### 3.1.2.8 Demographic information

We collected demographic information of participants including age, gender, race, ethnicity, highest level of education, whether they or any of their family members work in the fossil fuel industry, their zip code, and the year when they first started living within this zip code. We also measured political identity with two items. Participants first indicated which political party they identified with. They then indicated their political orientation on a seven-point scale where 1 = *very liberal*, 4 = *moderate*, and 7 = *very conservative*.

#### 3.1.3 Procedure

After giving informed consent, participants first completed the value orientation scale. Then, they read messages that described extreme weather events caused by climate change derived from the 2022 Intergovernmental Panel on Climate Change (IPCC) report (Tignor et al., 2022). Participants were randomly assigned to one of the three climate information conditions: control, proximal, or distal. Participants in all conditions read a passage describing how climate change has given rise to more frequent and severe extreme weather events that negatively impact people around the world. In the control condition, no additional information was given. In the proximal condition, Hurricane Imelda, which affected Texas in 2019, was given as an example of a natural disaster that intensified because of climate change. In the distal condition, Cyclone Idai, which affected Africa in 2019, was given as an example of a natural disaster that intensified because of climate change (see Supplementary material 2.7).

After reading the messages, participants completed a set of items evaluating their engagement with the information. Two items asked how much participants agreed that the information was easy to understand and credible. These items served to assess the equivalency of understandability and perceived credibility of information in different conditions, to prevent potential variations in these aspects from confounding our results. An open-ended question asked, “If you were to describe these paragraphs in your own words to a friend or family member, what would you say?” We used the open-ended response as an attention check and excluded participants who provided answers that were irrelevant to the passages or too vague to indicate that they read the paragraph carefully (e.g., single-word answers such as “weather” and “terrible”).

Next, participants completed the scales for risk perception, mitigation intention, policy support, psychological distance, efficacy perception, and personal experience of hurricanes. Finally, they completed a demographic survey. The demographic characteristics of the participants in Study 1 are shown in Table 1.

**Table 1:** Study 1: participant demographic characteristics.

Variable	*n*	%
Gender		
Female	318	67.4
Male	146	30.9
Other	6	1.3
Prefer not to say	1	0.2
Age		
18–24	55	11.7
25–34	76	16.1
35–44	88	18.6
45–54	64	13.6
55–64	82	17.4
65–74	69	14.6
75 or older	37	7.8
Race		
White or Caucasian	352	74.6
Black or African American	64	13.6
American Indian/Native American or Alaska Native	2	0.4
Asian	10	2.1
Native Hawaiian or other Pacific Islander	1	0.2
Other	15	3.2
Two or more races	27	5.7
Prefer not to say	2	0.4
Ethnicity		
Hispanic or Latinx	94	19.9
Do not identify as Hispanic or Latinx	377	79.9
Highest level of education		
Less than high school degree	28	5.9
High school graduate	126	26.7
Some college but no degree	121	25.6
Associate degree in college (2-year)	63	13.3
Bachelor’s degree in college (4-year)	88	18.6
Master’s degree or above, doctoral degree, or professional degree (JD, MD)	45	9.5
Political party		
Republican	155	32.8
Libertarian	7	1.5
Independent	140	29.7
Democrat	139	29.4
Other	29	6.1

### 3.2 Results

#### 3.2.1 Messaging condition did not affect psychological distance of climate change

**Figure 2:**
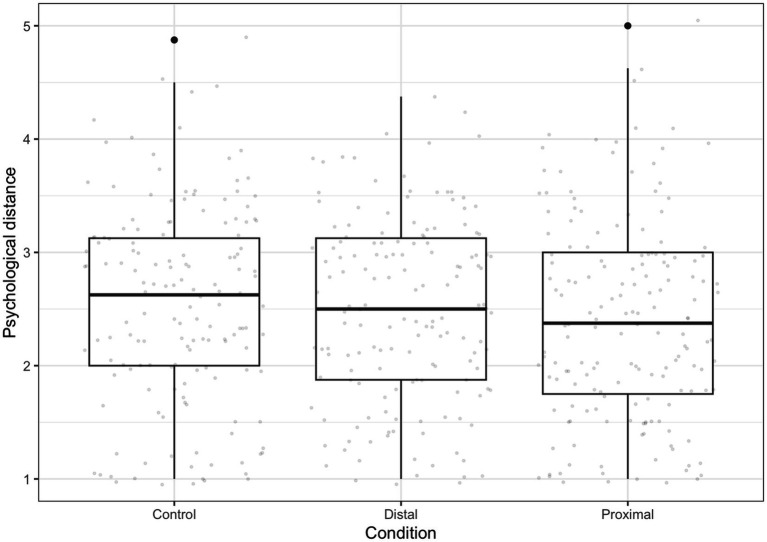
Psychological distance to climate change after proximal, distal, and control messaging conditions. The messaging conditions did not have a statistically significant effect on psychological distance. The average score around 2.5 indicates that, on average, individuals neither agree nor disagree that climate change is spatially and socially proximal to them.

We conducted a one-way between-subjects analysis of variance (ANOVA) to test the effect of messaging conditions on psychological distance of climate change. There was no significant difference in psychological distance between participants who read the control, distal, or proximal messages, *F*(2, 469) = 1.15, *ns* (see Figure 2).

#### 3.2.2 Self-transcendence, but not self-enhancement, predicted climate engagement

We performed Ordinary Least Squares (OLS) regressions to test whether messaging conditions interacted with values to affect climate engagement. We replicated the model used by Schoenefeld and McCauley (2016) and included messaging condition (dummy-coded with the control condition as the reference level), self-enhancement, self-transcendence, an interaction term between self-enhancement and condition, and another interaction term between self-transcendence and condition as predictors. We performed regressions with these predictors on risk perception, mitigation behavior, and policy support, respectively (see Table 2).

**Table 2:** Interaction effects of values and information condition on risk perception, mitigation behavior, and policy support.

Predictor	Risk perception	Mitigation intention	Policy support
(Intercept)	0.734 (0.465)	2.081*** (0.196)	1.575*** (0.300)
Self-transcendence	1.171*** (0.131)	0.222*** (0.055)	0.526*** (0.085)
Self-enhancement	−0.142 (0.104)	−0.018 (0.044)	−0.094 (0.068)
Distal message	0.154 (0.681)	−0.202 (0.287)	0.024 (0.440)
Proximal message	0.478 (0.653)	0.023 (0.275)	−0.220 (0.422)
Self-transcendence × Distal message	−0.069 (0.183)	0.070 (0.077)	−0.091 (0.118)
Self-transcendence × Proximal message	−0.141 (0.176)	−0.028 (0.074)	0.042 (0.114)
Self-enhancement × Distal message	0.096 (0.141)	−0.007 (0.059)	0.118 (0.091)
Self-enhancement × Proximal message	0.127 (0.144)	0.023 (0.061)	0.061 (0.093)
*N*	472	472	472
*R^2^*	0.357	0.128	0.226
logLik	−847.453	−439.520	−641.513
AIC	1714.905	899.039	1303.025

Each column shows the regression coefficients for the corresponding climate engagement outcome. Numbers in parentheses indicate standard errors. ***p < 0 0.001.

Results show that self-transcendence positively predicted all three dependent variables: risk perception, *b* = 1.17, *t*(463) = 8.95, *p* < 0.001, mitigation behavior, *b* = 0.22, *t*(463) = 4.02, *p* < 0.001, and policy support, *b* = 0.53, *t*(463) = 6.22, *p* < 0.001. This confirms our hypothesis that self-transcendence positively predicts risk perception, mitigation behavior, and policy support regardless of condition. However, contrary to our hypothesis, there was no significant interaction between messaging conditions and self-enhancement.

#### 3.2.3 Values interacted with psychological distance to predict climate engagement

While the messaging conditions did not significantly influence psychological distance to climate change, it is still possible that psychological distance interacted with values to affect climate engagement in the ways we hypothesized. To test this relationship, we conducted exploratory regression analyses where we replaced messaging conditions in the models above with psychological distance. We performed regressions with psychological distance, self-enhancement, self-transcendence, and interaction terms between psychological distance and values as predictors of risk perception, mitigation intention, and climate policy support, respectively (Table 3).

**Table 3:** Interaction effects of values and psychological distance on risk perception, mitigation behavior, and policy support.

Predictor	Risk perception	Mitigation intention	Policy support
(Intercept)	6.794*** (0.810)	1.889*** (0.404)	4.492*** (0.584)
Self-transcendence	0.335 (0.186)	0.360*** (0.093)	0.189 (0.134)
Self-enhancement	−0.175 (0.139)	−0.001 (0.069)	−0.271** (0.100)
Psychological Distance	−1.825*** (0.269)	0.085 (0.134)	−0.960*** (0.194)
Self-transcendence × Psychological Distance	0.121 (0.065)	−0.071* (0.032)	0.045 (0.047)
Self-transcendence × Psychological Distance	0.115* (0.052)	0.009 (0.026)	0.125*** (0.037)
*N*	472	472	472
*R* ^2^	0.562	0.168	0.343
logLik	−756.998	−428.559	−602.869
AIC	1527.995	871.118	1219.739

Each column shows the regression coefficients for the corresponding climate engagement outcome. Numbers in parentheses indicate standard errors. *p < 0 0.05. **p < 0 0.01. ***p < 0 0.001.

We found that closer psychological distance to climate change predicted higher risk perception, mitigation intention, and policy support. There was an interaction between self-enhancement and psychological distance, such that for individuals with stronger self-enhancement values, the effect of psychological distance on risk perception and policy support was weaker. There was an interaction between self-transcendence and psychological distance for mitigation intention in the opposite direction: For individuals with higher self-transcendence, the effect of psychological distance on mitigation intention was *stronger*.

## 4 Study 2: eco-anxiety and climate engagement

Because the proximal and distal messaging conditions in Study 1 did not significantly alter the psychological distance to climate change, we conducted Study 2 to examine whether an elicitation device to invoke eco-anxiety is more effective at increasing climate engagement. We preregistered the methods and analysis plan for Study 2 on the Open Science Framework (OSF).^2^

### 4.1 Materials and methods

The protocol for Study 2 was approved by the Institutional Review Board of Middlebury College (IRB #320). Participants volunteered to and gave consent to participating in the study. They were treated following APA ethical guidelines.

#### 4.1.1 Participants

Data were collected in April 2024. Through Prolific, we recruited 451 participants who were at least 18 years old and lived in Texas, U. S. A. at the time of the study. Prior to participating in the study, participants reported their gender and political leanings. We set sampling quotas based on gender and political leanings so that 25% of the whole sample was recruited from individuals who identified as politically conservative, 25% as moderate, 25% as liberal, and 25% as “other” or not defined by the previous options. Additionally, 50% of the whole sample was recruited from individuals who identified as male, and 50% as female. Participants received an average of $12 per hour in compensation for their participation in the study and were paid through Prolific. After excluding 37 participants who failed attention checks, the final sample included 414 participants.

#### 4.1.2 Measures

##### 4.1.2.1 Value orientation

Value orientation was measured using the Higher-Order-Value Scale-17 (HOVS17), a 17-item version of the Schwartz Value Survey (SVS) developed by Lechner et al. (2024) (see Supplementary material 2.8). The scale measures the four higher-order values theorized by Schwartz et al. (2012). Each value orientation subscale consists of at least three items. Example items include “It is important to her/him to show that her/his performance is better compared to the performance of other people.” Participants indicated how similar the person described in the item was to themselves on a fully labeled six-point rating scale where 1 = *is not at all similar to me* and 6 = *is very similar to me*. We computed the average of each subscale as an indicator of the corresponding higher-order value. The self-transcendence (α = 0.79) and the self-enhancement subscales (α = 0.77) both demonstrated good internal consistency.

##### 4.1.2.2 Eco-anxiety

Participants’ level of eco-anxiety was measured after the interventions using a scale (see Supplementary material 2.9) consisting of three subscales from the Hogg Eco-Anxiety Scale (HEAS) (Hogg et al., 2021): a four-item subscale assessing affective symptoms of anxiety, a three-item subscale assessing rumination, and a three-item subscale assessing anxiety about personal impact on the Earth. The fourth subscale in the HEAS, which assesses chronic behavioral symptoms related to climate anxiety (e.g., difficulty sleeping and enjoying social activities), is not included because chronic symptoms are unlikely to be affected immediately after the exercise. The HEAS has been validated in past research (Hogg et al., 2023; Innocenti et al., 2023) to have high internal consistency. Participants indicated the degree to which they experienced the symptoms at that moment on a 7-point Likert-style scale (1 = not at all, 7 = a great deal). We compute a single indicator of eco-anxiety by averaging scores across all items. A higher average score corresponds to higher levels of eco-anxiety. The eco-anxiety scale showed high internal consistency (α = 0.96).

##### 4.1.2.3 Risk perception

Consistent with Study 1, risk perception of climate change consequences was measured with four items on a 7-point scale that asked about participants’ concern about climate change and their believed severity and certainty of climate change impact. The risk perception scale showed high internal consistency (α = 0.94).

##### 4.1.2.4 Information sharing intention

Information sharing intention is defined as the intention to share information about climate change and to mitigate climate change through both personal behavior and supporting climate policy. To measure this, we developed a task modeled after Vlasceanu et al. (2024) where participants were given a short message about reducing carbon emissions through changing one’s food consumption pattern (see Supplementary material 2.10) and asked whether they would like to post the message to their social media accounts. Those who indicated their willingness to share were further asked where they had posted the information.

##### 4.1.2.5 Mitigation behavior

Behavior to mitigate climate change was elicited using an adapted version of the Work for Environmental Protection Task (Lange and Dewitte, 2022; see Supplementary material 2.11; henceforth, WEPT). In our version of the task, participants chose to exert the extra efforts to screen a set of 20 numerical stimuli in exchange for a $0.2 donation to the Texas Campaign for the Environment, a local environmental organization. Participants could screen a maximum of 5 sets of numbers. The total number of sets a participant chose to screen indicated their propensity of personal pro-environmental behavior. Lange and Dewitte (2023) found WEPT performance to be internally consistent and correlated to general pro-environmental behavior.

##### 4.1.2.6 Climate policy support

Consistent with Study 1, climate policy support was measured using four items derived from Schoenefeld and McCauley (2016) that asked participants to indicate their support for four different types of pro-environmental policies. The policy support scale showed high internal consistency (α = 0.82).

##### 4.1.2.7 Demographic information

Consistent with Study 1, we collected demographic information including age, gender, race, ethnicity, highest level of education, whether they or any of their family members work in the fossil fuel industry, their zip code, from what year they started living within this zip code, and their political identity.

#### 4.1.3 Procedure

Each participant completed an electronic survey distributed via Qualtrics. Participants first completed the HOVS-17 which measures their value orientation. They were then randomly assigned to one to the three eco-anxiety interventions (see Supplementary material 2.12). In the self-reflective writing condition, participants are asked to write about a past autobiographical event where climate change made them feel anxious in a text box. In the information exposure condition, participants are given a short passage about human causes of climate change and the Smokehouse Creek fire which happened in Texas in February 2024. After reading, they are asked to summarize the information in the passage in a text box. In the control condition, participants are asked to write about their evening routine in a text box.

Immediately after the intervention, participants completed a manipulation check where they rated their eco-anxiety. Then, they completed the risk perception scale, the information sharing task, the adapted WEPT which measured personal climate mitigating behavior, and the policy support scale. Finally, participants completed the questionnaire for demographic information. The demographic characteristics of the participants in Study 2 are shown in Table 4.

**Table 4:** Study 2: participant demographic characteristics.

Variable	*n*	%
Gender		
Male	195	47.1
Female	214	51.7
Other	5	1.2
Age (years old)		
18–24	54	13.0
25–34	133	32.1
35–44	109	26.3
45–54	58	14.0
55–64	35	8.5
65–74	20	4.8
75 or older	5	1.2
Race		
American Indian/Native American or Alaska Native	4	1.0
Asian	41	9.9
Black or African American	51	12.3
Native Hawaiian or Other Pacific Islander	1	0.2
White or Caucasian	273	65.9
Two or more races	20	4.8
Other	19	4.6
Prefer not to say	5	1.2
Ethnicity		
LatinX	96	23.2
Non-LatinX	318	76.8
Education		
Less than high school degree	4	1.0
High school graduate (or equivalent, including GED)	44	10.6
Attending some college	86	20.8
Associate degree in college (2-year)	45	10.9
Bachelor’s degree in college (4-year)	168	40.6
Master’s, doctoral, or professional degree (JD, MD)	67	16.2
Involvement in Fossil Fuel Industry		
None	372	89.9
Work in the industry	8	1.9
One or more family members work in the industry	34	8.2
Political Spectrum Identification		
Very liberal	55	13.3
Liberal	68	16.4
Somewhat liberal	47	11.4
Moderate	85	20.5
Somewhat conservative	63	15.2
Conservative	68	16.4
Very conservative	28	6.8

### 4.2 Results

#### 4.2.1 Self-reflective writing led to higher eco-anxiety than proximal messaging

**Figure 3:**
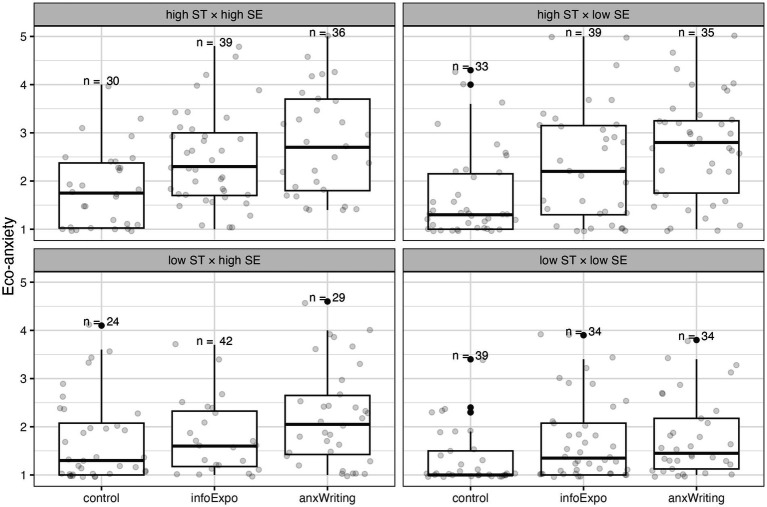
Eco-anxiety after the information exposure, self-reflective writing intervention, and control condition. Both interventions caused statistically significant increases in eco-anxiety. Further, participants who completed the self-reflective writing task experienced significantly higher eco-anxiety than those in the information exposure condition.

We conducted a one-way between-subjects ANOVA to test the effect of intervention conditions on eco-anxiety. The results are shown in Figure 3. There was a significant effect of intervention on eco-anxiety, *F*(2, 411) = 19.14, *p* < 0.01 (Supplementary Table S1a). Post-hoc tests showed that the self-reflective writing task significantly increased eco-anxiety when compared to proximal messaging, and proximal messaging significantly increased eco-anxiety when compared to control, supporting our first hypothesis (Supplementary Table S1b).

#### 4.2.2 Self-transcendence and eco-anxiety predicted climate engagement

We performed Ordinary Least Square (OLS) regressions to test for the main effects of eco-anxiety and values on four measures of climate engagement-risk perception, information sharing intention, mitigation behavior, and policy support, respectively. The regression coefficients are summarized in Table 5 (for complete results, see Supplementary Table S2). Self-transcendence positively predicted risk perception, *t*(414) = 5.687, *p* < 0.001, information sharing intention, *t*(339) = 2.692, *p* < 0.01, mitigation behavior, *t*(414) = 2.197, *p* < 0.05, and policy support, *t*(414) = 4.454, *p* < 0.001. Self-transcendence positively predicted all measures of climate engagement, consistent with our hypothesis and the findings in Study 1. Eco-anxiety also had significant positive effects on all four measures of pro-environmental behavior. Specifically, it positively predicted risk perception, *t*(414) = 15.031, *p* < 0.001, information sharing intention, *t*(339) = 6.243, *p* < 0.001, mitigation behavior, *t*(414) = 3.184, *p* < 0.01, and policy support, *t*(414) = 9.389, *p* < 0.001.

**Table 5:** Main effects of eco-anxiety and value orientation on climate engagement.

Predictor	Risk perception	Info. sharing intention	Mitigation behavior	Policy support
(Intercept)	0.331 (0.410)	−0.443** (0.140)	0.655 (0.690)	1.703*** (0.262)
Eco-anxiety	1.068*** (0.071)	0.158*** (0.025)	0.380** (0.119)	0.425*** (0.045)
Self-transcendence	0.454*** (0.080)	0.073** (0.027)	0.295 * (0.134)	0.227*** (0.051)
Self-enhancement	−0.037 (0.064)	0.041 (0.022)	−0.060 (0.108)	−0.005 (0.041)
*N*	414	339	414	414
*R* ^2^	0.460	0.181	0.049	0.269
logLik	−714.910	−195.163	−930.191	−528.894
AIC	1439.820	400.327	1870.382	1067.787

Info. sharing intention = Information sharing intention. Each column shows the regression coefficients for the corresponding climate engagement outcome. Numbers in parentheses indicate standard errors. *p < 0.05. **p < 0.01. ***p < 0 0.001.

#### 4.2.3 Eco-anxiety mediated the association between values and climate engagement

To establish the mechanism through which eco-anxiety and values predict climate engagement, we first performed separate OLS regressions to examine whether eco-anxiety moderated the association between self-transcendence, self-enhancement, and climate engagement. The regression coefficients are summarized in Table 6 (for complete results, see Supplementary Table S3). Contrary to our hypothesis, we found no significant interaction effect between eco-anxiety and self-transcendence, or between eco-anxiety and self-enhancement, on any of the climate engagement measures. This suggests that eco-anxiety did not moderate the association we found between self-transcendence values and climate engagement.

**Table 6:** Interaction effects of eco-anxiety with value orientations on pro-environmental behavior.

Predictor	Risk perception	Info. sharing intention	Mitigation behavior	Policy support
(Intercept)	−0.549 (0.908)	−0.496 (0.308)	−0.932 (1.524)	1.759** (0.580)
Eco-anxiety	1.565*** (0.464)	0.190 (0.161)	1.344 (0.778)	0.389 (0.296)
Self-transcendence	0.599*** (0.169)	0.084 (0.057)	0.707* (0.284)	0.207 (0.108)
Self-enhancement	0.016 (0.141)	0.041 (0.047)	−0.209 (0.237)	0.008 (0.090)
Eco-anxiety × Self-transcendence	−0.082 (0.083)	−0.006 (0.029)	−0.227 (0.139)	0.011 (0.053)
Eco-anxiety × Self-enhancement	−0.027 (0.057)	0.000 (0.019)	0.059 (0.095)	−0.006 (0.036)
*N*	414	339	414	414
*R* ^2^	0.462	0.181	0.057	0.269
logLik	−714.311	−195.140	−928.629	−528.860
AIC	1442.621	404.279	1871.258	1071.720

Info. sharing intention = Information sharing intention. Each column shows the regression coefficients for the corresponding climate engagement outcome. Numbers in parentheses indicate standard errors. *p < 0.05. **p < 0.01. ***p < 0 0.001.

Nevertheless, it is possible that values affected participants’ eco-anxiety in response to the intervention conditions, which in turn affected climate engagement. To test this, we constructed two binary variables for self-enhancement and self-transcendence where scores higher than or equal to the sample median were defined as “high” and otherwise as “low.” We conducted a 3 × 2 × 2 independent-samples ANOVA to examine how intervention, self-enhancement, and self-transcendence affected eco-anxiety (Table 7).

**Table 7:** Interaction effect of condition and value orientation on eco-anxiety (*N* = 414).

Variable	df	Sum sq.	Mean sq.	F value	*p* value
Condition	2	35.972	17.986	21.060	≤0.001***
Self-transcendence	1	29.401	29.401	34.426	≤0.001***
Self-enhancement	1	5.056	5.056	5.920	0.016*
Condition × Self-transcendence	2	5.166	2.583	3.014	0.050*
Condition × Self-enhancement	2	0.685	0.342	0.400	0.671
Self-transcendence × Self-enhancement	1	0.777	0.777	0.907	0.342
Condition × Self-transcendence × Self-enhancement	2	0.588	0.294	0.343	0.710
Residuals	402	344.440	0.857	NA	NA

*p < 0.05. **p < 0.01. ***p < 0 0.001.

**Figure 4:**
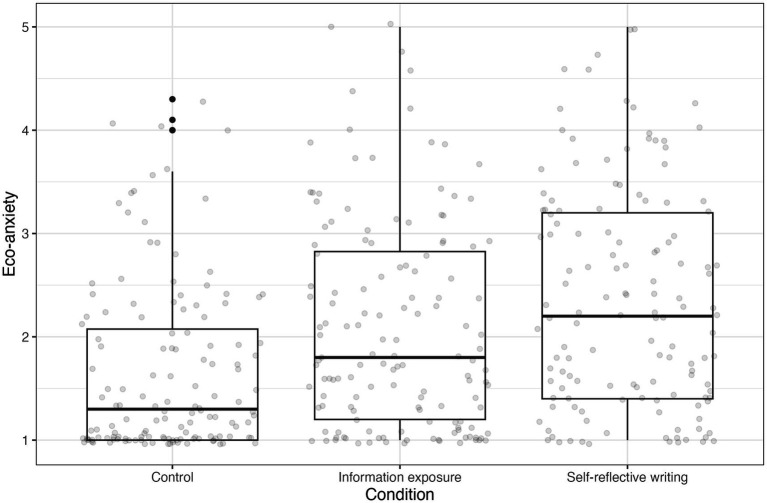
Eco-anxiety after each intervention condition, by value orientation types. Self-transcendence values are not associated with eco-anxiety in the control condition, but when given either intervention, participants with high self-transcendence values experienced significantly higher eco-anxiety than those with low self-transcendence values. For individuals with low self-transcendence, only self-reflective writing significantly increased eco-anxiety relative to control.

We found that eco-anxiety is significantly higher among participants with high self-transcendence, *F*(1, 412) = 34.426, *p* < 0.001. Eco-anxiety is also significantly higher among participants with high self-enhancement, *F*(1, 412) = 5.920, *p* < 0.05. Notably, we also found a significant interaction effect between condition and self-transcendence. As demonstrated in Figure 4, self-transcendence values are not associated with the level of eco-anxiety in the control condition, but when given either intervention, participants with high self-transcendence values experienced significantly higher eco-anxiety than those with low self-transcendence values. For individuals with low self-transcendence, only self-reflective writing significantly increased eco-anxiety relative to control. The *post hoc* analyses in Supplementary Table S4 support these observations. They suggest that values not only directly predict climate engagement but also influence the level of eco-anxiety in response to climate communication interventions, which then affects engagement behaviors.

**Figure 5:**
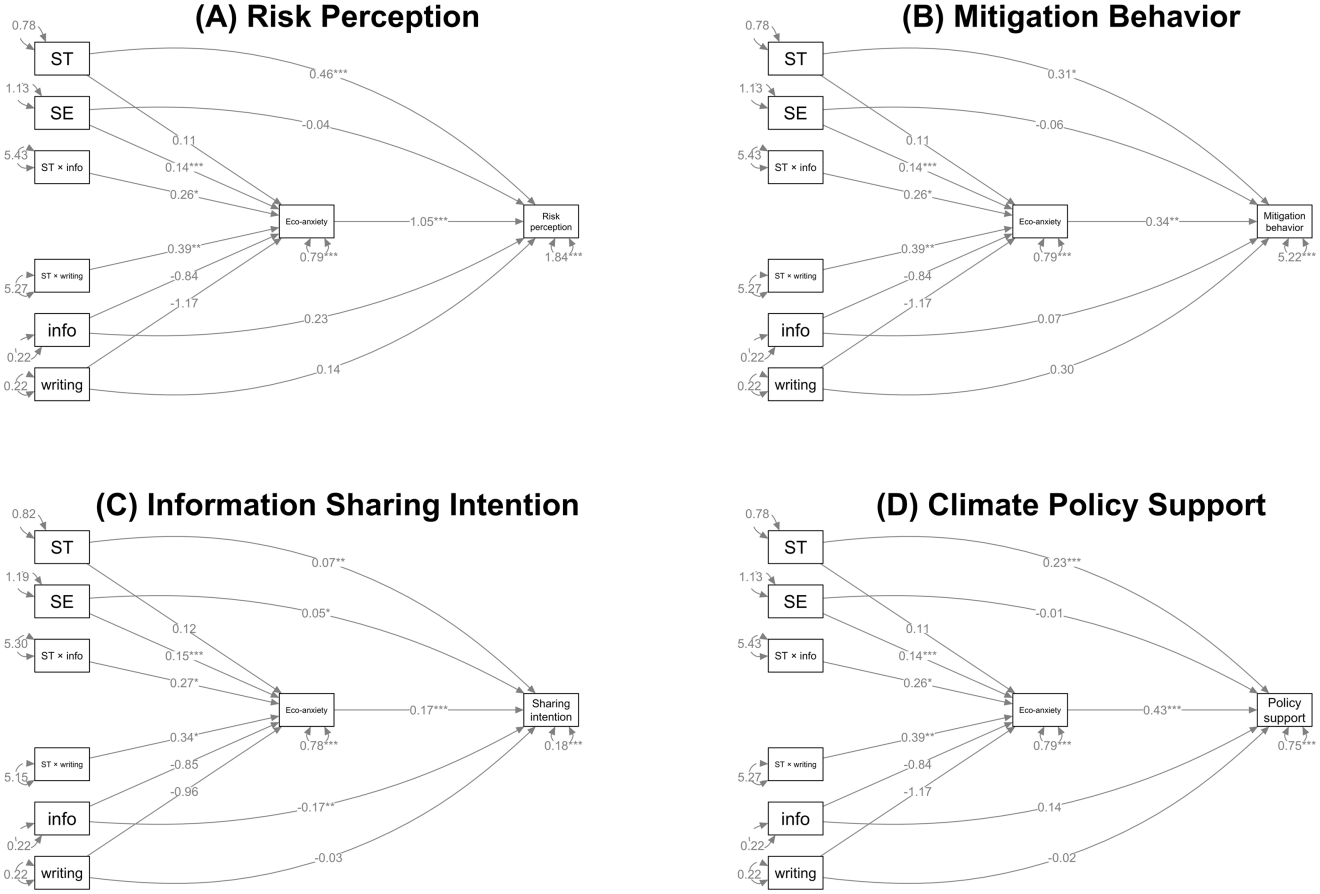
Path diagrams for the effect of value orientation, intervention, and eco-anxiety on the four measures of climate engagement outcomes. ST = Self-transcendence; SE = Self-enhancement; info = information exposure; writing = self-reflective writing. The diagrams are constructed based on path analysis, and the results support that values and the intervention jointly determine climate engagement. Self-transcendence directly predicted increases in risk perception **(A)**, mitigation behavior **(B)**, information sharing intention **(C)**, and policy support **(D)**. Among those, it predicted the strongest increase in risk perception, followed by policy support. On the other hand, self-enhancement directly predicted an increase in information sharing **(C)** but only indirectly predicted the other measures through eco-anxiety. Both interventions only significantly increased risk perception, mitigation behavior, and policy support through increasing eco-anxiety; However, the proximal messaging directly led to a decline in information sharing intention, despite increasing eco-anxiety which encouraged information sharing.

Based on these results, we conducted path analyses to model how value orientation, intervention, and eco-anxiety may affect the four measures of climate engagement outcomes (Figure 5). All four models showed good fit to the data (see Supplementary Table S5 for the model fit indices). We found that self-transcendence directly predicted increases in all four measures of climate engagement, while self-enhancement only directly predicted an increase in information sharing intention. Self-transcendence also predicted larger increases in eco-anxiety in response to the interventions. In turn, of all measures of climate engagement, eco-anxiety led to the strongest increase in risk perception, followed by policy support, mitigation behavior, and information sharing intention. The models also support that eco-anxiety explains the effect of interventions on climate engagement: both interventions only significantly increased risk perception, mitigation behavior, and policy support through increasing eco-anxiety. However, information sharing intention is an exception to this trend: proximal messaging directly led to a decline in information sharing intention, despite increasing eco-anxiety which encouraged information sharing.

#### 4.2.4 Eco-anxiety boosted risk perception and policy support among conservative individuals

Since we did not observe a backlash in climate engagement in response to high eco-anxiety among participants with strong self-enhancement values, we further tested if high eco-anxiety inhibited climate engagement among politically conservative participants. First, analysis of correlations between political orientation, values, demographic characteristics, and climate engagement (Table 8) shows that conservatism is negatively correlated with eco-anxiety, policy support, risk perception, info sharing intention, and mitigation behavior. Additionally, conservatism increased with age and was weakly positively correlated with working in the fossil fuel industry.

**Table 8:** Correlations of political orientation with value orientation, behavioral outcomes, and demographic characteristics.

Variable	r	*p* value
Self-enhancement	0.085	0.086
Self-transcendence	−0.282	≤0.001***
Condition	−0.078	0.113
Eco-anxiety	−0.406	≤0.001***
WEPT behavior	−0.107	0.030*
Policy support	−0.625	≤0.001***
Risk perception	−0.588	≤0.001***
Info. sharing intention	−0.278	≤0.001***
Age	0.232	≤0.001***
Gender	−0.069	0.163
Education	−0.020	0.685
Work in fossil fuel	0.130	0.008**

Political orientation is coded such that higher values denote a stronger conservative orientation; As such, a positive correlation indicates that conservative individuals are more likely to score high on this variable. To ensure statistical power and facilitate interpretation, work in fossil fuel is coded such that 1 = oneself or a family member works in the fossil fuel industry and 0 = otherwise. *p < 0.05. **p < 0.01. ***p < 0 0.001.

We further tested whether political orientation predicted climate engagement in addition to the level of eco-anxiety. We conducted OLS regressions to test whether eco-anxiety interacts with political orientation to affect the four measures of climate engagement (Table 9). There is a positive interaction effect between conservatism and eco-anxiety on risk perception and policy support, despite a negative main effect of conservatism on both outcomes. This indicates that high eco-anxiety caused significantly larger increases in risk perception and policy support among more politically conservative participants than less conservative ones.

**Table 9:** Interaction effect of eco-anxiety and political orientation on perception and behavior.

Predictor	Risk perception	Info. sharing intention	Mitigation behavior	Policy support
(Intercept)	4.380*** (0.474)	−0.231 (0.192)	0.573 (0.954)	4.163*** (0.299)
Self-transcendence	0.320*** (0.068)	0.062* (0.027)	0.283* (0.137)	0.131** (0.043)
Self-enhancement	0.004 (0.055)	0.052* (0.023)	−0.042 (0.111)	0.04 (0.035)
Eco-anxiety	0.131 (0.121)	0.141** (0.049)	0.47 (0.244)	−0.072 (0.076)
Political orientation	−0.801*** (0.070)	−0.035 (0.028)	0.034 (0.142)	−0.468*** (0.044)
Eco-anxiety × Political Orientation	0.203*** (0.031)	−0.003 (0.013)	−0.033 (0.062)	0.094*** (0.019)
*N*	414	339	414	414
R^2^	0.623	0.202	0.05	0.503
logLik	−640.544	−190.753	−929.943	−449.009
AIC	1295.089	395.506	1873.887	912.018

Political orientation is coded such that higher values denote a stronger conservative orientation. *p < 0.05. **p < 0.01. ***p < 0 0.001.

Finally, we conducted OLS regressions to identify factors that predicted high eco-anxiety among politically conservative participants. The self-reflective writing intervention, as well as both self-enhancement and self-transcendence, positively predicted eco-anxiety among politically conservative individuals (Table 10).

**Table 10:** Effect of value orientation and demographic characteristics on eco-anxiety among politically conservative individuals (*N* = 159).

Term	Estimate	Std.error	Statistic	*p* value
(Intercept)	−0.02	0.35	−0.05	0.957
Condition_infoExposure	0.13	0.14	0.89	0.377
Condition_anxietyWriting	0.46	0.14	3.36	≤0.001***
Self-enhancement	0.25	0.06	4.48	≤0.001***
Self-transcendence	0.17	0.06	2.67	0.008**
Age	−0.08	0.04	−1.84	0.068
Gender	−0.08	0.11	−0.7	0.482
Education	0.06	0.04	1.35	0.179
Work in fossil fuel	−0.12	0.15	−0.77	0.443

To ensure statistical power and facilitate interpretation, work in fossil fuel is coded such that 1 = oneself or a family member works in the fossil fuel industry and 0 = otherwise. *p < 0.05. **p < 0.01. ***p < 0 0.001.

## 5 Discussion

Through two experimental studies, we examine how value orientation interacts with proximal information and eco-anxiety to affect climate engagement. Study 1 (N = 472) tested how exposure to spatially proximal versus distal messaging affected self-reported psychological distance and subsequent climate risk perception, policy support, and mitigation intentions. We found that spatial messaging conditions did not significantly affect psychological distance to climate change or climate engagement. However, both self-transcendence values and proximal psychological distance directly predicted more climate engagement. Additionally, the association between proximal psychological distance and pro-environmental behavior was stronger among individuals with higher self-transcendence and weaker among individuals with higher self-enhancement. Study 2 (N = 414) examined whether a self-reflective writing task to evoke eco-anxiety is more effective than proximal messaging at increasing climate engagement. We found that the writing task was more effective than proximal messaging at eliciting eco-anxiety, which positively predicted risk perception, policy support, information sharing intention, and effortful mitigation behavior. Stronger self-transcendence values not only directly predicted more climate engagement but also predicted higher eco-anxiety in response to either intervention. Collectively, our results suggest that elicitation of eco-anxiety could be a superior strategy for increasing engagement with climate change than exposure to information about its local impacts.

### 5.1 Self-enhancement predicted eco-anxiety but not climate engagement

In line with previous research (e.g., Boto-García and Bucciol, 2020; Schoenefeld and McCauley, 2016; Steg et al., 2014), results from both of our studies support that individuals with stronger self-transcendence values are more likely to perceive the risks posed by climate change as severe, share information about climate change on social media, support pro-climate policies, and take personal actions to mitigate climate change. Additionally, while most previous works measured mitigation behavior through participants’ self-reports of their past or intended actions, Study 2 administered a task where participants screened numbers in exchange for donation to an environmental organization to provide concrete evidence that stronger self-transcendence values are associated with more effortful mitigation behavior.

Our findings reveal a much more complex relationship between self-enhancement and climate engagement. In Study 1, we did not find the effect of proximal messaging to vary by the level of self-enhancement values, but the positive association between psychological proximity to climate change and climate engagement was weaker among individuals with stronger self-enhancement values. While we did not replicate Schoenefeld and McCauley’s (2016) finding that proximal messaging reduced pro-environmental behavior among individuals with high self-enhancement values when compared to either distal or control messaging, our results still support the authors’ conjecture that psychological proximity to climate change causes reactance among individuals with high self-enhancement.

One possible reason why self-enhancement interacted with psychological distance but not the messaging conditions in Study 1 is that the messages did not successfully alter participants’ psychological distance of climate change. This failure to manipulate psychological distance could be due to a floor effect: our participants reported a very proximal psychological distance to climate change overall (*M* = 2.47, *SD* = 0.87), with 74.4% reporting a psychological distance of 3 out of 5 or closer. This psychological proximity is consistent with the increase of severe weather and climate disasters over the past decade (Smith, 2024). Nearly half (44.7%) of the participants in Study 1 had personally experienced a hurricane and the levels of damage experienced from hurricanes were significantly correlated with closer psychological distance. Alternatively, the floor effect could also be due to the lack of a true “no-message” condition. While Schoenefeld and McCauley (2016) did not provide any information to their control group, the control group in Study 1 read a passage about climate change where distance is not implied to make sure the conditions differ only in the spatial proximity of the described climate disaster. It is possible that, compared to no information, even a control message can reduce the psychological distance of climate change by increasing its salience. Unfortunately, we could not test this assumption due to the lack of a “no-message” condition for comparison. Taken together, the results from Study 1 show that individuals with high self-enhancing values, a growing proportion of which have already experienced the impact of climate change locally, are unlikely to increase pro-environmental behavior in response to messages that highlight the spatial proximity of climate change.

On the other hand, in Study 2, we found evidence that stronger self-enhancement values were associated with increased eco-anxiety and was an even stronger predictor of eco-anxiety than self-transcendence among politically conservative individuals. One potential explanation for this is that because individuals with high self-enhancement tend to prioritize their own interests, they are more attuned to the threats that climate change poses towards their personal well-being, regardless of their political beliefs. While research has highlighted that individuals with high self-enhancement values responded more positively to messages that appeal to the financial benefits of pro-environmental behavior (Birkenbach and Egloff, 2024), to our knowledge, the present study is among the first to demonstrate the link between self-enhancement and higher levels of anxiety towards climate change. This finding suggests that the lower levels of pro-environmental behavior among individuals with high self-enhancement may not be due to a lack of concern as proposed in some previous works (e.g., Schultz et al., 2005), but rather a disjunction between concern and behavior.

In line with this, the path analysis in Study 2 reveals that higher self-enhancement values directly predicted a higher likelihood of sharing a short message about mitigating climate change on social media, but not other less public forms of climate engagement. Research documenting the link between self-enhancement values and information sharing online has suggested that self-enhancement motivates people to present a positive self-image by seeking and transmitting positive self-relevant information (Zheng et al., 2020). It is possible that individuals with higher self-enhancement values are more likely to forward information on social media in general, or they perceive sharing information about climate change as beneficial for their self-image. This unique link between self-enhancement and information sharing points to the importance of designing public, community-based forms of participation for turning self-enhancement into a motivation for pro-environmental action. Self-enhancement may encourage, rather than inhibit, pro-environmental behavior when collective norms, interests, and goals result in an inner obligation to become actively involved (Bamberg et al., 2015).

### 5.2 Personal experiences more effectively increased engagement than external information

Whereas messaging did not significantly alter psychological distance in Study 1, Study 2 points to eliciting personal experiences that invoke eco-anxiety as an effective strategy for encouraging pro-environmental behavior across individuals with diverse value orientations. The self-reflective writing task led to higher levels of eco-anxiety, which positively predicted subsequent pro-environmental behavior, than messaging about the local impacts of climate change. While previous literature has shown that incidental emotions influence support for climate policy (Lu and Schuldt, 2015) and personal experiences of climate change increase mitigation behavior (Haden et al., 2012), our finding offers robust evidence that cued recall of climate-related experiences can increase climate policy support and personal mitigation behavior through invoking eco-anxiety. As the areas and communities worldwide directly affected by climate change continue to grow, messaging campaigns that call on personal experiences will become vital for encouraging pro-environmental behavior.

Importantly, self-enhancement values did not mediate the pathway from self-reflective writing to eco-anxiety or that from eco-anxiety to climate engagement. This suggests that recalling personal, anxiety-inducing experiences does not cause the reactance that individuals with strong self-enhancement display towards external information about the local impacts of climate change. This might simply be because recalling a personal experience leaves a much smaller scope for psychologically distancing oneself from the impact and urgency of climate change (McDonald et al., 2015) than reading the message about a local wildfire. Alternatively, the message highlighted the scale and damage of the wildfire in aggregate, which could have undermined the participants’ sense of efficacy in mitigating the impacts of climate change through their personal actions and thus discourage them from acting pro-environmentally.

The path analysis in Study 2 aligns with this conjecture, as the information exposure condition directly reduced the subsequent intention to share a post about mitigating climate change by reducing one’s own meat and dairy consumption, despite indirectly increasing it through increasing eco-anxiety. It is possible that, besides causing eco-anxiety, exposure to the aggregate scale and severity of a climate disaster also undermined the belief that one could mitigate climate change through their own behavior. Although we did not include a post-intervention measure of self-efficacy in Study 2, which would allow for a direct test of this conjecture, we collected written summaries of the message from participants exposed to it and written recollections of their experience from those who engaged in self-reflection. In the future, we plan to conduct thematic analysis of these text responses to examine whether exposure to the local effects of climate change undermined self-efficacy or changed other climate-related beliefs when compared to reflection on personal experiences of climate change.

### 5.3 Limitations and future research directions

Several limitations warrant mention. First, most of the respondents to Study 1 (67.4%) were women. This gender imbalance could be because women are often more likely to respond to surveys than men (Dunn et al., 2004; Kalmijn and Liefbroer, 2011), and the overrepresentation of women in Study 1 may have contributed to the high engagement with climate change we observed (Brink and Wamsler, 2019). Because we focused on how value orientation affects climate engagement, and gender was not correlated with values, we did not control for gender in our analyses to ensure statistical power. Nevertheless, future research should account for the potential effects of gender on climate engagement. Second, responses to eco-anxiety can be both adaptive, such as a sense of responsibility and increased engagement, and maladaptive, such as denial (Léger-Goodes et al., 2022). It is possible that our measures did not account for the potential negative impacts of eco-anxiety on mental health and individuals’ engagement with climate change.

Despite these limitations, our work offers the following novel contributions to research and practice: First, we measured post-intervention levels of psychological distance to climate change and eco-anxiety, allowing us to empirically test mechanisms through which local information and personal experiences affect pro-environmental behavior. Second, the experimental design in Study 2 enabled distinguishing the effects of eco-anxiety in reaction to the intervention from those of pre-existing eco-anxiety and trait anxiety. Third, our results demonstrate that cued recall of personal, anxiety-inducing experiences of climate change could be a more effective strategy for increasing pro-environmental behavior than exposure to information about the proximal effects of climate change; For individuals with strong self-enhancement values, our findings suggest that public, community-based forms of participation may also turn self-enhancement into a motivation for pro-environmental action.

## 6 Conclusion

Through two experimental studies, this paper provides empirical evidence on the role of value orientations in climate decision-making and the effectiveness of increasing pro-environmental behavior through influencing eco-anxiety. Study 1 shows that messages highlighting the spatial proximity of climate impacts did not influence psychological distance of climate change and were not effective interventions for climate engagement; Nonetheless, self-reported psychological distance predicted climate engagement. This correlation was less pronounced for individuals with high self-enhancement values. Study 2 shows that a self-reflective writing task was more effective than proximal messaging at eliciting eco-anxiety, which positively predicted risk perception, policy support, information sharing intention, and effortful mitigation behavior. Stronger self-transcendence values not only directly predicted more climate engagement but also predicted higher eco-anxiety in response to either intervention. Collectively, our findings suggest that elicitation of eco-anxiety could be a superior strategy for increasing engagement with climate change than exposure to information about its local impacts. They also prompt future research to explore interventions that possibly evoke more personal experience of climate impacts, as well as how their effects vary depending on one’s value orientation.

## Data Availability

The datasets presented in this study can be found in online repositories. The names of the repository/repositories and accession number(s) can be found at: https://github.com/hanwzhang/valuesAndClimateEngagement.

## Footnotes

^1^ https://osf.io/f5vew/?view_only=1d9eb0c926674b9ba04f101730bc8c00

^2^ https://osf.io/6jb98/?view_only=e16aa74b4d4e44ca974e5d2c58f87d76
