# Supplementary material for: Values interact with psychological distance and eco-anxiety to promote climate engagement: insights from two experimental studies
Source: Front Psychol. 2025 Sep 19;16:1646889. doi: 10.3389/fpsyg.2025.1646889 (PMC12491825; doi:10.3389/fpsyg.2025.1646889)
Supplement: Supplementary file 1 [file Supplementary_file_1.docx]

Supplementary Material

# Supplementary Tables

**Table S1a. Effect of intervention conditions on eco-anxiety.**

| Variable | df | sumsq | meansq | statistic | p.value |
| --- | --- | --- | --- | --- | --- |
| condition | 2 | 35.97 | 17.99 | 19.15 | ≤.001*** |
| Residuals | 411 | 386.11 | .94 | NA |  |

**Table S1b. Post-hoc tests on the effect of intervention conditions on eco-anxiety.**

| Condition | diff | lwr | upr | p adj |
| --- | --- | --- | --- | --- |
| control-anxWriting | -.72 | -.99 | -.44 | ≤.001*** |
| InfoExpo-anxWriting | -.28 | -.55 | -.00 | 0.046* |
| InfoExpo-control | .44 | .17 | .71 | ≤.001*** |

**Table S2. Main effects of eco-anxiety and values on pro-environmental behavior**

|  | estimate | std.error | statistic | p.value |
| --- | --- | --- | --- | --- |
| Risk perception |  |  |  |  |
| (Intercept) | 0.331 | 0.41 | 0.808 | 0.42 |
| ECO-ANXIETY | 1.068 | 0.071 | 15.031 | ≤.001*** |
| SELF-TRANSCENDENCE | 0.454 | 0.08 | 5.687 | ≤.001*** |
| SELF-ENHANCEMENT | -0.037 | 0.064 | -0.581 | 0.562 |
| Info. sharing intention |  |  |  |  |
| (Intercept) | -0.443 | 0.14 | -3.165 | 0.002** |
| ECO-ANXIETY | 0.158 | 0.025 | 6.243 | ≤.001*** |
| SELF-TRANSCENDENCE | 0.073 | 0.027 | 2.692 | 0.007** |
| SELF-ENHANCEMENT | 0.041 | 0.022 | 1.851 | 0.065 |
| Mitigation behavior |  |  |  |  |
| (Intercept) | 0.655 | 0.69 | 0.95 | 0.343 |
| ECO-ANXIETY | 0.38 | 0.119 | 3.184 | 0.002** |
| SELF-TRANSCENDENCE | 0.295 | 0.134 | 2.197 | 0.029* |
| SELF-ENHANCEMENT | -0.06 | 0.108 | -0.561 | 0.575 |
| Policy support |  |  |  |  |
| (Intercept) | 1.703 | 0.262 | 6.51 | ≤.001*** |
| ECO-ANXIETY | 0.425 | 0.045 | 9.389 | ≤.001*** |
| SELF-TRANSCENDENCE | 0.227 | 0.051 | 4.454 | ≤.001*** |
| SELF-ENHANCEMENT | -0.005 | 0.041 | -0.123 | 0.902 |

**Table S3. Interaction effects of eco-anxiety and values on pro-environmental behavior**

|  | estimate | std.error | statistic | p.value |
| --- | --- | --- | --- | --- |
| Risk perception |  |  |  |  |
| (Intercept) | -0.549 | 0.908 | -0.605 | 0.546 |
| Eco-anxiety | 1.565 | 0.464 | 3.377 | ≤.001*** |
| Self-transcendence | 0.599 | 0.169 | 3.545 | ≤.001*** |
| Self-enhancement | 0.016 | 0.141 | 0.112 | 0.911 |
| Eco-anxiety × Self-transcendence | -0.082 | 0.083 | -0.989 | 0.323 |
| Eco-anxiety × Self-enhancement | -0.027 | 0.057 | -0.472 | 0.637 |
| Info. sharing intention |  |  |  |  |
| (Intercept) | -0.496 | 0.308 | -1.612 | 0.108 |
| Eco-anxiety | 0.19 | 0.161 | 1.177 | 0.24 |
| Self-transcendence | 0.084 | 0.057 | 1.468 | 0.143 |
| Self-enhancement | 0.041 | 0.047 | 0.864 | 0.388 |
| Eco-anxiety × Self-transcendence | -0.006 | 0.029 | -0.216 | 0.829 |
| Eco-anxiety × Self-enhancement | 0 | 0.019 | -0.007 | 0.995 |
| Mitigation behavior |  |  |  |  |
| (Intercept) | -0.932 | 1.524 | -0.612 | 0.541 |
| Eco-anxiety | 1.344 | 0.778 | 1.728 | 0.085 |
| Self-transcendence | 0.707 | 0.284 | 2.49 | 0.013* |
| Self-enhancement | -0.209 | 0.237 | -0.883 | 0.378 |
| Eco-anxiety × Self-transcendence | -0.227 | 0.139 | -1.631 | 0.104 |
| Eco-anxiety × Self-enhancement | 0.059 | 0.095 | 0.623 | 0.533 |
| Policy support |  |  |  |  |
| (Intercept) | 1.759 | 0.58 | 3.033 | 0.003** |
| Eco-anxiety | 0.389 | 0.296 | 1.314 | 0.19 |
| Self-transcendence | 0.207 | 0.108 | 1.918 | 0.056 |
| Self-enhancement | 0.008 | 0.09 | 0.093 | 0.926 |
| Eco-anxiety × Self-transcendence | 0.011 | 0.053 | 0.201 | 0.841 |
| Eco-anxiety × Self-enhancement | -0.006 | 0.036 | -0.156 | 0.876 |

**Table S4. Post-hoc Tests on the interaction effect of condition and self-transcendence on eco-anxiety**

| Condition × ST | | 1. – (2) | lwr | upr | p.adj |
| --- | --- | --- | --- | --- | --- |
| (1) | (2) |  |  |  |  |
| control × low ST | control × high ST | -0.23 | -0.68 | 0.22 | 0.702 |
| infoExpo × low ST | infoExpo × high ST | -0.62 | -1.07 | -0.17 | 0.001** |
| anxWriting × low ST | anxWriting × high ST | -0.75 | -1.21 | -0.3 | ≤.001*** |
| infoExpo × high ST | control × high ST | 0.62 | 0.18 | 1.06 | 0.001** |
| anxWriting × high ST | control × high ST | 0.98 | 0.53 | 1.43 | ≤.001*** |
| anxWriting × high ST | infoExpo × high ST | 0.36 | -0.08 | 0.81 | 0.184 |
| infoExpo × low ST | control × low ST | 0.22 | -0.23 | 0.68 | 0.722 |
| anxWriting × low ST | control × low ST | 0.45 | 0 | 0.91 | 0.048* |
| anxWriting × low ST | infoExpo × low ST | 0.23 | -0.23 | 0.69 | 0.701 |

**Table S5. Goodness of fit indices of the path analysis models**

|  | Chi sq. | CFI | RMSEA |
| --- | --- | --- | --- |
| Risk perception | 4.29 | .99 | .05 |
| Info. sharing intention | 3.79 | .99 | .05 |
| Mitigation behavior | .64 | 1.00 | .00 |
| Policy support | 1.54 | 1.00 | .00 |

Note. Chi sq.: Chi square, CFI: Comparative Fit Index, RMSEA: Root Mean Square Error of Approximation

# Supplementary Materials

## Shortened Schwartz Value Survey (15-item)

From Stern et al. (1998)

*Please rate the importance of the following values as guiding principles in your life.*

*Use the 7-point scale, where -1 indicates that the value is opposed to your principles, 0 indicates that the value is not important for you, and 5 indicates that the value is extremely important to you.*

1. Protecting the environment, preserving nature

2. Unity with nature, fitting into nature

3. Respecting the earth, harmony with other species

4. A world at peace, free of war and conflict

5. Social justice, correcting injustice, care for the weak

6. Equality, equal opportunity for all

7. Honoring parents and elders, showing respect

8. Family security, safety for loved ones

9. Self-discipline, self-restraint, resistance to temptation

10. Authority, the right to lead or command

11. Influential, having an impact on people and events

12. Wealth, material possessions, money

13. A varied life, filled with challenge, novelty, and change

14. An exciting life, stimulating experiences

15. Curious, interested in everything, exploring

## Psychological Distance Scale

Adapted from Wang et al. (2019)

*Please indicate how much you agree to the following statements on the scale: 1 = “Strongly disagree” to 5 = “Strongly agree”.*

**Spatial distance:**

1. I feel geographically far from the effects of climate change.

2. Serious effects of climate change will mostly occur in areas far away from here.

3. My local area will be affected by climate change.

4. Climate change will have consequences for every region, including where I live.

**Social distance:**

5. I don’t see myself as someone who will be affected by climate change.

6. Serious effects of climate change will mostly affect people who are distant from me.

7. My family and I will be safe from the effects of climate change.

8. I can identify with victims of climate related disasters.

## Climate Risk Perception Scale

Adapted from Chu & Yang [(2020)](https://www.zotero.org/google-docs/?broken=y1WGKq)

Participants reported their perception of the risk of climate change.

*Please indicate your answers to the following questions on the scale: 1 = “Not at all concerned/serious/likely/important” to 7 = “Very concerned/serious/likely/important”.*

1. How concerned are you about climate change?

2. How serious are the current impacts of climate change?

3. How likely do you think that you will personally experience a negative impact due to climate change in the next five years?

4. How important is climate change to you in relation to other political and social issues?

## Personal Mitigation Intention

Adapted from Wang et al. (2019)

*We are interested to know about the real choices that people make in day-to-day life. How likely are you to do the following things in the next six months? There are no right or wrong answers. (1 = very unlikely, 2 = unlikely, 3 = undecided, 4 = likely, 5 = very likely)*

1. Buy a regular shampoo for $5, compared to an eco-friendly shampoo for $10? ____
2. Buy organic or local vegetables at an average cost of $30 per week, compared to

imported vegetables at an average cost of $20 per week? ____

1. Catch a bus somewhere for 20 minutes, rather than driving there for 5 minutes? ____
2. Walk to the shops for 15 minutes, rather than driving there for 3 minutes? ____
3. Throw recyclable materials in a general waste bin, rather than hold onto recyclable

material until there is a recycle bin nearby? ____

1. Leave appliances turned on at power outlets on the wall, rather than switch off

appliances at the wall when not in use? ____

1. Take a 5-minute shower instead of a 10-minute shower to cut down on water use? ____

## Climate Policy Support Scale

Adapted from Schoenefeld & McCauley [(2016)](https://www.zotero.org/google-docs/?broken=h0lbci)

Participants indicated their support of the following government policy proposals.

*There are a number of government policies that might be used to help the U.S. shift to renewable energy (e.g., wind, solar, biomass). How much do you support the following policies?*

*(response scale: 1= Definitely do not support the policy; 2 = Probably not support the policy; 3 = Undecided; 4 = Probably support the policy; 5 = Definitely support the policy)*

1. Paying individual households directly (a subsidy) to use renewable energy in their homes.

2. Stopping or reducing government funding (subsidies) for coal, oil, and natural gas companies.

3. Increasing government funding (subsidies) for renewable energy companies.

4. A cap-and-trade system, where companies would receive a certain amount of permits (a budget) to emit carbon dioxide each year. If their annual emissions exceed their budget, they would have to buy more permits from companies that emitted less than their budget.

## Efficacy Perception

From Chu & Yang (2020)

*Please indicate how much you agree with the following statements on the scale: 1 = “Strongly disagree” to 5 = “Strongly agree”.*

1. I believe my actions can have a beneficial influence on climate change.

2. Actions I take personally can help reduce the impacts of climate change.

3. Climate change can be averted by mobilizing collective effort.

4. If we act collectively, we will be able to minimize the consequences of climate change.

## Study 1 Messaging Conditions

**Control**

*Please carefully read the following paragraph and answer the subsequent questions.*

Some extreme weather events are becoming more frequent and severe as a result of climate change, according to the *Climate Change 2022: Impacts, Adaptation and Vulnerability* report published by the United Nations Intergovernmental Panel on Climate Change (IPCC).^^[[1]](#footnote-1)^^ These include extremely heavy rainfall, extreme and prolonged heat leading to catastrophic fires, and more frequent and severe hurricanes and cyclones. These disasters have led to loss of lives and damage to biodiversity, health, infrastructure, and the economy.

**Proximal**

*Please carefully read the following paragraphs and answer the subsequent questions.*

Some extreme weather events are becoming more frequent and severe as a result of climate change, according to the *Climate Change 2022: Impacts, Adaptation and Vulnerability* report published by the United Nations Intergovernmental Panel on Climate Change (IPCC). These include extremely heavy rainfall, extreme and prolonged heat leading to catastrophic fires, and more frequent and severe hurricanes and cyclones. These disasters have led to loss of lives and damage to biodiversity, health, infrastructure, and the economy.

One example of such disasters is Hurricane Imelda, which struck Texas in 2019. The hurricane led to widespread and devastating flooding that affected millions of people, causing serious economic impact and a significant number of deaths. The unprecedented destructive impact of Hurricane Imelda was largely caused by climate change. Researchers have found that Imelda was more intense, and storms like Imelda are more likely to happen, because of climate change.

**Distal**

*Please carefully read the following paragraphs and answer the subsequent questions.*

Some extreme weather events are becoming more frequent and severe as a result of climate change, according to the *Climate Change 2022: Impacts, Adaptation and Vulnerability* report published by the United Nations Intergovernmental Panel on Climate Change (IPCC). These include extremely heavy rainfall, extreme and prolonged heat leading to catastrophic fires, and more frequent and severe hurricanes and cyclones. These disasters have led to loss of lives and damage to biodiversity, health, infrastructure, and the economy.

One example of such disasters is Cyclone Idai, which struck the African continent in 2019. The cyclone led to widespread and devastating flooding that affected millions of people, causing serious economic impact and a significant number of deaths. The unprecedented destructive impact of Cyclone Idai was largely caused by climate change. Researchers have found that climate change has caused more intense rainfall associated with tropical cyclones.

## The Higher-Order-Value Scale-17 (HOVS17)

| **Item** | **Wording (English)** | **Basic value** |
| --- | --- | --- |
| **Conservation** | | |
| **12CO** | It is important to her/him to obey all laws. | Conformity (rules) |
| **7CO** | It is important to her/him that the state is strong and can defend its citizens. | Security (societal) |
| **4CO** | It is important to her/him to maintain traditional values and ways of thinking. | Tradition |
| **Openness to Change** | | |
| **3OC** | It is important to her/him to develop her/his own opinions. | Self-direction (thought) |
| **8OC** | It is important to her/him to expand her/his knowledge. |  |
| **14OC** | It is important to her/him to be free to choose by herself/himself what s/he does. | Self-direction (action) |
| **17OC** | It is important to her/him to figure things out herself/himself. |  |
| **10OC** | It is important to her/him to have all kinds of new experiences. | Stimulation |
| **Self-Enhancement** | | |
| **2SE** | It is important to her/him to show that her/his performance is better compared to the performance of other people. | Achievement |
| **15SE** | It is important to her/him that people recognize what s/he achieves. |  |
| **6SE** | It is important to her/him to be rich. | Power (resources) |
| **11SE** | It is important to her/him to be the one who tells others what to do. | Power (dominance) |
| **Self-Transcendence** | | |
| **9ST** | It is important to her/him to help the people dear to her/him. | Benevolence (caring) |
| **13ST** | It is important to her/him to concern herself/himself with every need of her/his dear ones. |  |
| **1ST** | It is important to her/him to care for nature. | Universalism (nature) |
| **5ST** | It is important to her/him to be tolerant toward all kinds of people and groups. | Universalism (tolerance) |
| **16ST** | It is important to her/him that everyone be treated justly, even people s/he doesn’t know. | Universalism (concern) |

[(Lechner et al., 2024)](https://www.zotero.org/google-docs/?broken=FIXewa) Notes: The item labels refer to the item’s order in the inventory (number) and the higher-order value it measures (letter). For example, 12CO is the 12th item and measures conservation.

## The Eco-anxiety Scale

Adapted from the Hogg Eco-Anxiety Scale (HEAS) [(Hogg et al., 2021)](https://www.zotero.org/google-docs/?broken=bVHhhw)

Participants indicated how much they identify with the following experiences (response scale: 1 = not at all, 5 = a great deal).

*How often have you been bothered with the following problems, when thinking about climate change?*

1. *Feeling nervous, anxious or on edge*
2. *Not being able to stop or control worrying*
3. *Worrying too much*
4. *Feeling afraid*
5. *Unable to stop thinking about future climate change and other global environmental problems*
6. *Unable to stop thinking about past events related to climate change*
7. *Unable to stop thinking about losses to the environment*
8. *Feeling anxious about the impact of your personal behaviors on the earth*
9. *Feeling anxious about your personal responsibility to help address environmental problems*
10. *Feeling anxious that your personal behaviors will do little to help fix the problem*

## Information Sharing Intention

Adapted from Vlasceanu et al. [(2024)](https://www.zotero.org/google-docs/?broken=U6wNdv)

Participants read the short message below and answered the following multiple choice questions.

*Did you know that removing meat and dairy for only two of three meals per day could decrease food-related carbon emissions by 60%? It is an easy way to fight #ClimateChange source:* [*https://econ.st/3qjvOnn*](https://econ.st/3qjvOnn)

*Are you willing to share this information on your social media?* Choices:

- *Yes, I am willing to share this information*
- *No, I am not willing to share this information*
- *I do not use social media*

[Follow-up, if willing to share] *On what platform(s) will you post the information?* Choices:

- *Facebook*
- *X (previously known as Twitter)*
- *Instagram*
- *Other (text entry)*

## The adapted Work for Environmental Protection Task (WEPT)

Adapted from Lange and Dewitte [(2022)](https://www.zotero.org/google-docs/?broken=sQoWrp)

Example stimulus:

*In the following, we will ask you if you want to complete additional pages of the number-identification task.*

*For each page that you correctly complete, we will make a $0.2 donation to Texas Campaign for the Environment, an environmental non-profit organization empowering Texas to fight pollution. Texas Campaign for the Environment campaigns, for example, to protect Texas' coastal bend and to stop desalination pollution in the area. It is up to you to decide how much time and effort you want to invest in the task.*

*There are a maximum of 5 pages that you can complete.*

1. *For each page, you will have to check 20 numbers.*
2. *You can decide, for each page separately, if you want to do this additional effort or not.*

*Doing this task is completely voluntary. If you want, you can decline checking the numbers (by selecting 'no') and go directly to the next part of the study. Continue on to your first page.*

*The next page will contain 20 numbers and we will donate $0.2 for the protection of Texas’ coastal bend if you complete this page. If you decide to complete this page, please do so thoroughly because we can only count pages that are at least 80% correct. We will not give you feedback, so please check whether your answers are correct before proceeding to the next page.*

***Do you want to complete this page?***

- *Yes*
- *No*

***Task 1 of 5***

*Identify all those stimuli with an even first digit and an odd second digit.*

*For example, you should click on '25', because the first digit (2) is even and the second digit is odd (5).*

*28 85 13 49 94 81 70 65 25 47 26 46 71 17 32 70 65 30 99 54*

## Study 2 interventions

Participants were exposed to one of the three following tasks.

**Self Reflective Writing.** Adapted from Lu and Schuldt [(2015)](https://www.zotero.org/google-docs/?broken=RjH4jH)

*We would like you to describe in detail one situation where climate change makes you (or has made you) anxious. This could be something you are presently experiencing or something from the past. Begin by writing down what you remember of the anxiety-inducing event and continue by writing as detailed a description of it as possible. If you can, please write your description so that someone reading this might even get angry just from learning about the situation.*

**Exposure to Information.** Adapted from Vlasceanu et al. [(2024)](https://www.zotero.org/google-docs/?broken=d6nD6n); Stimulus from Sheriff [(2024)](https://www.zotero.org/google-docs/?broken=TqzNtO)

*Please carefully read the following paragraph and answer the subsequent questions.*

*There is no doubt that humans are the main driver of climate change. Human influence has warmed the atmosphere, ocean, and land. Climate change is already affecting every region across the world. It has resulted in more frequent and intense extreme weather events, causing widespread harm and damage to people, wildlife, and ecosystems. Human systems are being pushed beyond their ability to cope and adapt.*

*One such example is the Smokehouse Creek fire, which started on 26 February in Hutchinson County, Texas. The worst wildfire in Texas’ history, it has so far burned more than 1.2 million acres (486,000 hectares) and killed two people and thousands of cattle. On 27 February, Texas Governor Greg Abbott issued a disaster declaration for 60 counties in response to the wildfire. Wildfire risk is expected to increase across Texas as climate change brings drier, hotter conditions, according to a 2021 report by Texas state climatologist John Nielsen-Gammon. The wildfire season will likely last longer in places where there is little rain, such as eastern Texas and areas commonly affected by wildfires may expand eastward, as fuels become drier faster, thanks to a warmer climate.*

*Now, we would like you to summarize, in a few sentences, the information in the text you just read. Please try to write down as many details as you can remember.*

**Control.** Adapted from Lu and Schuldt [(2015)](https://www.zotero.org/google-docs/?broken=qIk010)

*We would like you to describe in detail how you typically spend your evening. Begin by writing down a list of activities and then figure out how much time you devoted to each activity. Examples of things you might describe include eating dinner, hanging out with certain friends, watching TV, studying, etc. If you can, please write your description so that someone reading this might be able to reconstruct the way in which you, specifically, spend your evenings.*

1. <https://www.ipcc.ch/report/ar6/wg2/downloads/report/IPCC_AR6_WGII_FinalDraft_FullReport.pdf> [↑](#footnote-ref-1)
